# Pgu-Face: A dataset of partially covered facial images

**DOI:** 10.1016/j.dib.2016.09.002

**Published:** 2016-09-09

**Authors:** Seyed Reza Salari, Habib Rostami

**Affiliations:** a Electrical Engineering Department, School of Engineering, Persian Gulf University, Bushehr, Iran; b Computer Engineering Department, School of Engineering, Persian Gulf University, Bushehr, Iran

**Keywords:** Facial image, Partially covered facial images, Age estimation, Facial authentication

## Abstract

In this article we introduce a human face image dataset. Images were taken in close to real-world conditions using several cameras, often mobile phone׳s cameras. The dataset contains 224 subjects imaged under four different figures (a nearly clean-shaven countenance, a nearly clean-shaven countenance with sunglasses, an unshaven or stubble face countenance, an unshaven or stubble face countenance with sunglasses) in up to two recording sessions. Existence of partially covered face images in this dataset could reveal the robustness and efficiency of several facial image processing algorithms. In this work we present the dataset and explain the recording method.

**Specifications Table**

**Table:** 

Subject area	*Computer Science*
More specific subject area	*Image processing, Face recognition, Facial age estimation*
Type of data	*Image, table*
How data was acquired	*Images were taken using several mobile phone cameras and other commercial cameras*
Data format	*Raw, analyzed*
Experimental factors	*We captured four face images from any 224 subjects in two sessions with different figures. Images were taken in different conditions. Two sessions separated by minimum six days time.*
Experimental features	*We collected 896 face images with multiple cameras resolutions (4 images per subjects) and different sizes.*
Data source location	Tropical regions of the southwest of Iran
Data accessibility	*Data is publicly available on mendeley data public repository with 10.17632/znpyrgbfdr.1 doi, at*http://dx.doi.org/10.17632/znpyrgbfdr.1

**Value of the data**

• Images of this new dataset can be used for evaluating facial image processing systems such as face recognition or facial age estimation under partially occlusion, especially that it contains four different facial occlusion.
• Upper and lower face occlusion is one the main specific of this new dataset. Some facial image algorithms work well on lower face occlusion while not well for upper occlusion. Face upper regions contain the most human identity information, then upper occlusion covers these information [1]. This new dataset covers upper occlusion in addition to lower occlusion, and therefore can be used to evaluate facial image processing performance on the both regions.
• Age of each subject was provided in a text file with subject images, therefore it suitable for showing robustness of age estimation systems against occlusion problem.

## 1 Data

The Pgu-Face dataset contains 896 images from 224 different subjects. All of the subjects were Iranian men and most of them live in tropical regions of the southwest of Iran. The age range of the subjects was 16–82 years with average 27.89 years.

## 2 Experimental design, materials and methods

### 2.1 Data collection method

**Fig. 1:**
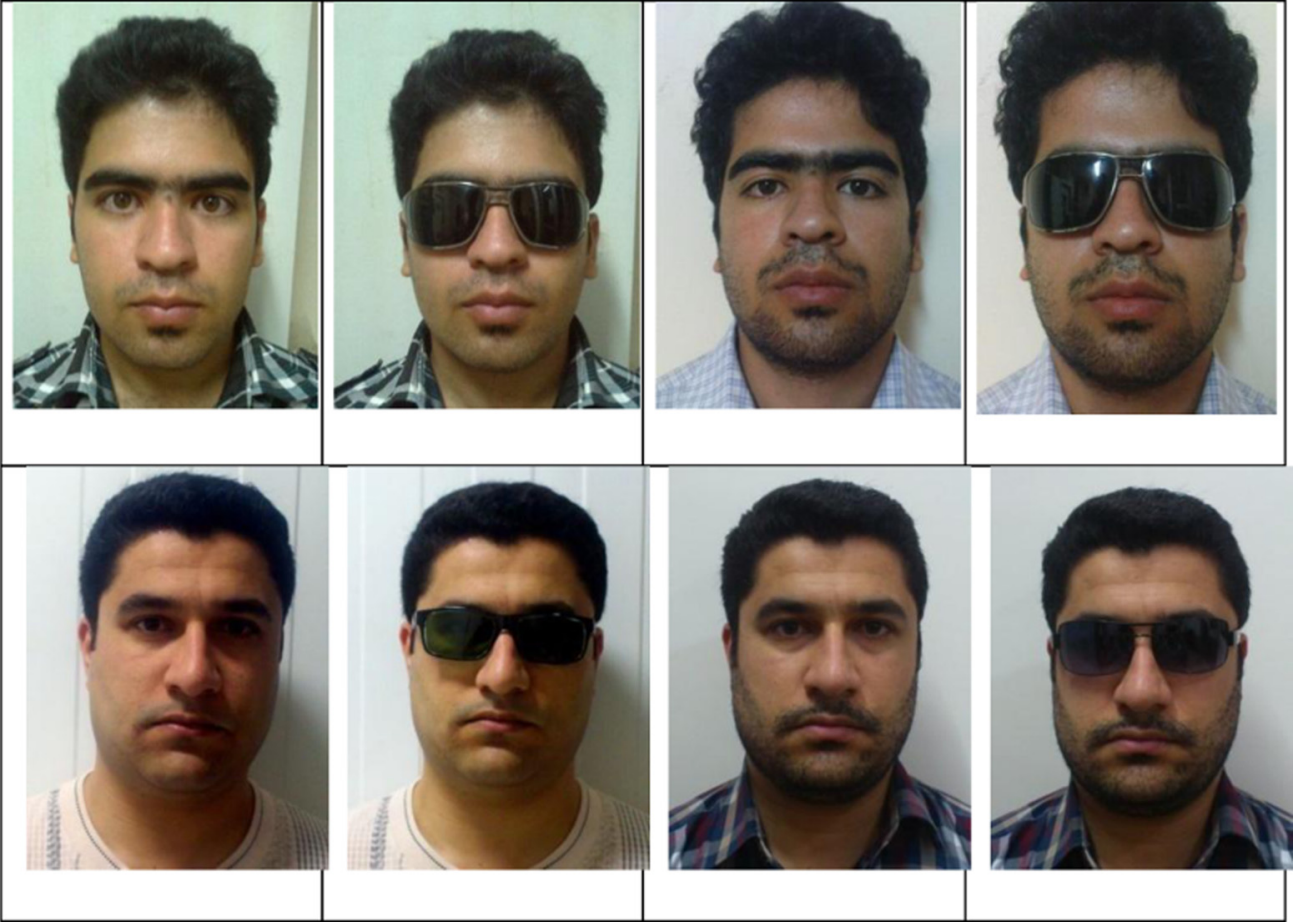
Example image׳s of two subject׳s in the four different countenance.

We captured four face images from each subject in two sessions with different figures. During each session, we recorded two neutral frontal images. In the first session, for each person, two images were recorded in such a way that in an image the subject׳s face was not covered by any cover such as a beard, mustache, glasses, etc., and was in a nearly clean-shaven countenance. In another image, the subject wore sunglasses. In the next session, for each subject, two images were recorded, including an image of the subject׳s face covered only with a beard and mustache, and then in another image the subject wore sunglasses. The minimum period between these two sessions was six days. Images were taken from about fifty to one hundred centimeters and subject׳s faces were in neutral expression without any specific angle to the camera. The images were taken in different locations, where the locations often were roofed. No specific camera stands were applied to position cameras. The light at each location was natural and most of the images were captured at night. For every subject, as described before, images were denominated as img01 to img04, respectively. Table 1 lists four different subject׳s figures. Fig. 1 shows all images from two subjects for two sessions.

### 2.2 Resolution of images

The resolution of the used cameras varies in range of 2–26 mega pixels. Therefore, the images dimensions varied over the used cameras. Most of the utilized cameras were commercial and mobile phone cameras. Mobile phone cameras against professional cameras have a lower quality and hence were suitable for our purpose, although they may have a better performance versus surveillance cameras. No necessary settings for all cameras were applied and all of the recorded images were captured in conventional conditions. The resolution of used cameras in addition to the number of images in each resolution were shown in Table 2. Based on achieved experimental results, it can deduced that the existence of facial occlusion, such as glasses, beards and mustache on the face, decrease probability of recognition and reliability of system. Hence, Pgu-Face dataset can be used to challenge recently presented facial image processing algorithms.

## Figures and Tables

**Table 1:** Four different subject׳s countenance images recorded in two sessions. (two sessions were separated with 6 day minimum time between them).

	Session 1	Session 2
First image	In a nearly clean-shaven countenance	In a stubble or unshaven face countenance
Second image	In a nearly clean-shaven countenance with sunglasses	In a stubble or unshaven face countenance with sunglasses

**Table 2:** Resolution of image׳s and number of image׳s in each resolution. (It is may be mentioned that in some of resolutions, multiple mobile phone camera brands were used, however, have a same resolution in mega pixels).

Resolution in mega pixels	Less than 5	5	8	10	12	13	14.1	16	20	26
Number of images	142	242	342	12	14	64	4	4	60	12
