# Supplementary material for: Pgu-Face: A dataset of partially covered facial images
Source: Data Brief. 2016 Sep 9;9:288–91. doi: 10.1016/j.dib.2016.09.002 (PMC5024140; doi:10.1016/j.dib.2016.09.002)
Supplement: Supplementary file 2 — Supplementary material [file mmc2.doc]

# CONFLICT OF INTEREST STATEMENT

***The Journal of Arthroplasty***

(Adopted from the American Academy of Orthopaedic Surgeons disclosure statement)

The following form **must be filled out completely and submitted by each author (example, 6 authors, 6 forms). If no discloser is required, please write/type “none” at the end of each sentence.**

Manuscript Title

1. Royalties from a company or supplier (The following conflicts were disclosed)

2. Speakers bureau/paid presentations for a company or supplier (The following conflicts were disclosed)

3A. Paid employee for a company or supplier (The following conflicts were disclosed)

3B. Paid consultant for a company or supplier (The following conflicts were disclosed)

3C. Unpaid consultants for a company or supplier (The following conflicts were disclosed)

4. Stock or stock options in a company or supplier (The following conflicts were disclosed)

5. Research support from a company or supplier as a Principal Investigator (The following conflicts were disclosed)

6. Other financial or material support from a company or supplier (The following conflicts were disclosed)

7. Royalties, financial or material support from publishers (The following conflicts were disclosed)

8. Medical/Orthopaedic publications editorial/governing board (The following conflicts were disclosed)

9. Board member/committee appointments for a society (The following conflicts were disclosed)

**Each author must sign AND print or type his/her name, date and submit a separate form**

In addition, one BLINDED Conflict of Interest form (no author names used) should be submitted per manuscript with all author disclosures.

Author Name (Seyed Reza Salari) Author Signature S.R.Salari Date: July 18, 2016
